# A Biochemical Analysis of Patients with COVID-19 Infection

**DOI:** 10.1155/2021/1383830

**Published:** 2021-10-22

**Authors:** Adil R. Sarhan, Thaer A. Hussein, Mohammed H. Flaih, Khwam R. Hussein

**Affiliations:** Department of Medical Laboratory Techniques, Nasiriyah Technical Institute, Southern Technical University, Nasiriyah 64001, Iraq

## Abstract

Several studies have demonstrated that age, comorbidities, and abnormalities in different clinical biomarkers can be important to understand disease severity. Although clinical features of COVID-19 have been widely described, the assessment of alterations of the most common biochemical markers that are reported in patients with COVID-19 still has not been well established. Here, we report clinical and blood biochemical indicators of 100 patients with COVID-19. Throat-swab upper respiratory samples were obtained from patients and real-time PCR was used to confirm SARS-CoV-2 infection. Gender, age, and clinical features such as diabetes mellitus, hypertension, and smoking habits were investigated. Biochemical parameters were categorized and analyzed according to these clinical characteristics. Triglycerides, GPT, and ALP are the biochemical markers that changed the most in the group of hypertension patients. Cholesterol and triglycerides were significantly different (*P*=0.01; *P*=0.04, respectively) between diabetic and nondiabetic patients with COVID-19. Potassium levels were significantly different (*P*=0.03) when comparing smokers with nonsmoker patients. Our results suggest several potential biochemical indexes that changed in patients with COVID-19 and whether certain comorbidity and clinical characteristics influence these markers.

## 1. Introduction

Coronavirus disease 2019 (COVID-19) is a contagious disease caused by the recently discovered severe acute respiratory syndrome coronavirus 2, which is still spreading throughout the world. Most infected patients with COVID-19 will have mild to moderate respiratory illness that will be recovered without special medical care. However, elderly people and those with chronic medical conditions such as respiratory disease, heart disease, diabetes, and cancer are more likely to develop severe illnesses [1–3]. According to recent World Health Organization (WHO) figures, more than 110 million cases have been confirmed worldwide, with 2.44 million deaths as of February 16, 2021 [4].

Iraq has been greatly impacted by the ongoing outbreak of COVID-19. The first COVID-19 patient was reported in Iraq on 24 February 2020 [5]. Currently, as of February 16, 2021, over 650,000 diagnosed cases have been reported with more than 13,000 deaths [4]. Several biochemical tests are dramatically changed in patients with COVID-19. Early studies of COVID-19 showed substantial levels of alanine aminotransferase (ALT) in intensive care unit patients [6]. In addition, D-dimer, creatinine, blood urea, and neutrophil levels were also substantially increased in patients with severe symptoms, while lymphocyte counts were reduced [7]. D-dimer levels were also elevated in 70% of patients with severe symptoms and deceased cases [8].

Deng et al. found that, in certain patients, ALT and aspartate transaminase (AST) showed higher levels than the normal range with decreased total bilirubin levels. Creatinine and creatine phosphokinase were both increased in 13% of patients with COVID-19 [9] while Wu et al. observed a change in the percentages of liver function biomarkers (ALB, GGT, AST, ALT, TBIL, and ALP) [10].

This study aimed to demonstrate a systematic assessment for some of the biochemical laboratory tests in patients with COVID-19. All patients were admitted to the Al-Hussein Teaching Hospital, Thi-Qar Province, Iraq. Real-time PCR was used to confirm SARS-CoV-2 infection. Clinical characteristics and blood biochemical tests of COVID-19 patients were examined and recorded.

## 2. Materials and Methods

### 2.1. Data Collection

All patients were referred to Al-Hussein Teaching Hospital in Thi-Qar Province, Iraq, between November 2 and December 30, 2020, showing COVID-19 symptoms. Throat-swab upper respiratory specimens were obtained from 100 patients and real-time PCR (polymerase chain reaction) was used to confirm SARS-CoV-2 infection. Clinical characteristics and blood biochemical tests of COVID-19 patients were examined and recorded. Gender, age, and clinical characteristics such as diabetes mellitus, hypertension, and smoking have been investigated. Informed consent was obtained from patients. The study has been approved by the Institutional Review Board.

### 2.2. Sample Collection and Data Processing

Venous blood (4.5 mL) was obtained. Blood samples were dispensed into a gel tube. All tubes were allowed to stand for 30 minutes at room temperature, followed by centrifugation for 10 minutes at 3500 rpm to get the serum. Liver and kidney function tests including alanine transaminase (ALT), aspartate aminotransferase (AST), alkaline phosphatase (ALP), total bilirubin, creatinine, and blood urea were measured. Uric acid, triglyceride, total cholesterol, high-density lipoprotein (HDL), calcium (Ca^+2^), sodium (Na^+^), potassium (K^+^), chloride (Cl^−^), magnesium (Mg^+2^), and phosphorus (P) were also measured using Gesan Chem-200 platform (Gesan Production SRL, Italy) according to the manufacturing protocols.

### 2.3. Statistical Analysis

Biochemical parameters were analyzed and categorized according to the following variables: (a) diabetes mellitus, (b) blood pressure, (c) smoking, and (d) gender. Biochemical tests were compared across patients grouped in these categories. Significance testing was performed in GraphPad Software using a *t*-test. A *P* value of <0.05 indicated statistical significance.

## 3. Results

A total of 100 COVID-19 patients were admitted to Al-Hussein Teaching Hospital in Thi-Qar Province, Iraq, from 2 November to 30 December 2020. Most cases (52%) were between the ages of 21 and 40 years, followed by those aged 41–60 years (29%). Approximately 54% of patients were female and 46% were male (Table 1). Most cases experienced influenza-like symptoms such as fever, cough, and mild myalgia during their time at the hospital. All patients were discharged following recovery of clinical symptoms. Diabetes mellitus, present in 23 out of 100 patients, was the most common comorbidity, followed by blood pressure, present in 14 patients. In addition, a summary of biochemical indexes outcomes among patients with COVID-19 is presented in Table 2.

**Figure 1:**
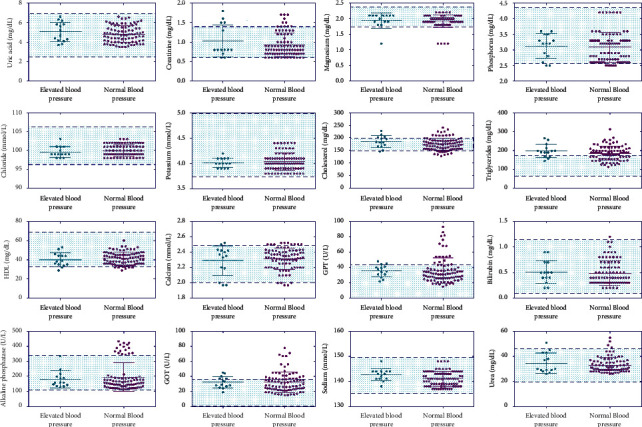
Scatter plots showing all the laboratory biochemical tests analyzed according to COVID-19 patients with normal blood pressure versus elevated blood pressure. The highlighted area indicates the normal range of each test.

**Figure 2:**
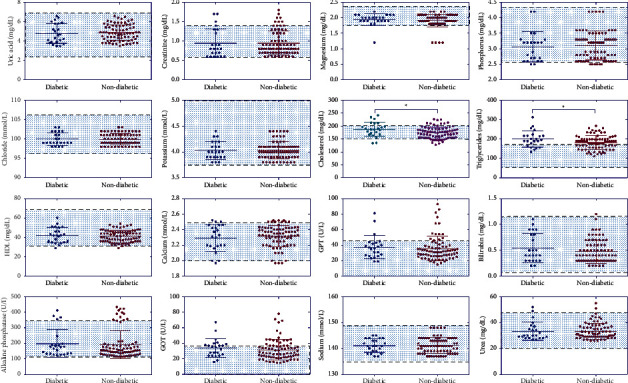
Scatter plots showing all the laboratory biochemical tests analyzed according to COVID-19 patients with diabetic versus nondiabetic. The highlighted area indicates the normal range of each test. Statistical significance was calculated by *t*-test (^*∗*^*P* < 0.05).

In the blood pressure group, triglycerides, alanine aminotransferase (GPT), and alkaline phosphatase (ALP) were the most common biochemical laboratory abnormalities identified (Figure 1). However, no significant association was found between elevated blood pressure and normal blood pressure in all biochemical laboratory parameters as shown in Figure 1. We then analyzed the correlation between the biochemical characteristics and diabetes mellitus in patients with COVID-19. Among the biochemical parameters, cholesterol and triglycerides had a significant difference (*t* = 2.572, *P*=0.01; *t* = 1.992, *P*=0.04, respectively) between the diabetic and nondiabetic COVID-19-infected patients (Figure 2). Furthermore, in a few patients, creatinine, alanine aminotransferase (GPT), alkaline phosphatase (ALP), and aspartate aminotransferase (GOT) levels were shown to be higher than the normal range. However, when comparing them according to diabetic and nondiabetic classification, none of these variations were statistically significant (Figure 2).

**Figure 3:**
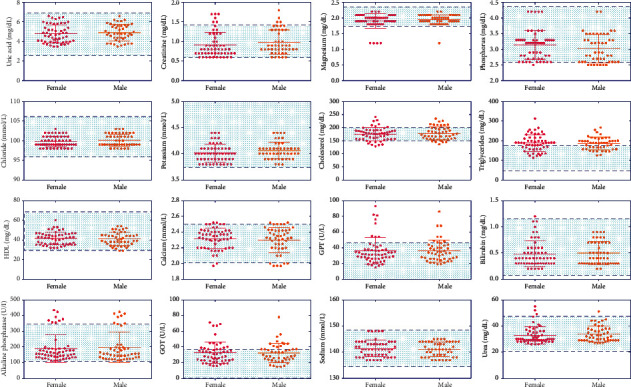
Scatter plots showing all the laboratory biochemical tests analyzed according to gender. The highlighted area indicates the normal range of each test.

Figure 3 indicates the gender-based breakdown of biochemical indexes. Out of 100 patients, 54% of the patients were female and 46% were male. Some biochemical measures such as creatinine, triglycerides, alkaline phosphatase and GOT, were found to have increased levels (Figure 3). However, when comparing these biomarkers between males and females, no evidence of significant differences was found.

**Figure 4:**
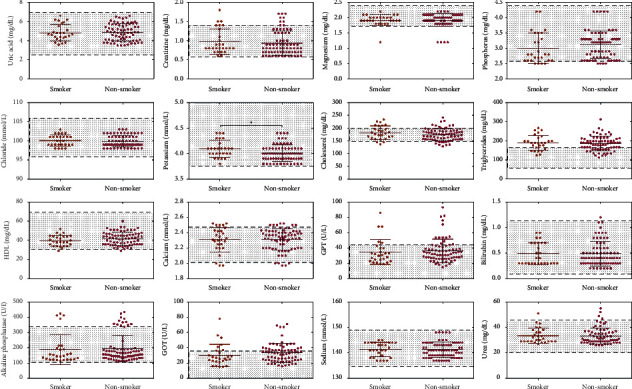
Scatter plots showing all the laboratory biochemical tests analyzed according to smoking habits. The highlighted area indicates the normal range of each test. Statistical significance was calculated by *t*-test (^*∗*^*P* < 0.05).

Smoking has been shown to increase serum lipid profiles including triglycerides [11, 12]. We, therefore, analyze whether smoking habits may influence the balance of serum biochemistry in patients with COVID-19. Low serum concentrations of magnesium, phosphorus, and calcium, have been seen in certain patients (Figure 4). Further analysis revealed high serum levels of creatinine, GPT, ALP, GOT, and urea in just a few other patients (Figure 4) whereas only potassium concentrations were significantly different (*t* = 2.140, *P*=0.03) when comparing smokers with nonsmoker patients.

## 4. Discussion

COVID-19 is an ongoing pandemic and the virus is still spreading worldwide. As stated by World Health Organization (WHO) reports, more than 110 million cases have been confirmed globally and 2.44 million deaths through February 16, 2021 [4]. On 24 February 2020, the first COVID-19 patient was confirmed in Iraq [5]. Recently, as of February 16, 2021, over 650,000 diagnosed cases have been reported with over 13,000 deaths [4]. Several studies have shown that age, comorbidities, and abnormalities of various clinical biomarkers can be essential to understand disease severity [13–16]. Even though clinical features of COVID-19 have been widely described, the overview of changes in the most common biochemical parameters that are observed in patients with COVID-19 infection is still unclear. Therefore, this report aims to study the changes in certain biochemical markers encountered in patients with COVID-19. In addition, clinical characteristics and comorbidity in 100 patients with COVID-19 have also been studied.

A strong relationship between lipid profiles and hypertension has been reported in [17–20]. Higher triglycerides values were found in patients with elevated blood pressure which are consistent with these reports. We found that triglycerides, GPT, and GOT were elevated although there was no significant difference between COVID-19 patients with elevated and normal blood pressure suggesting that COVID-19 infection may alter these biochemical laboratory markers regardless of hypertension. Furthermore, the higher triglyceride levels in certain patients might be due to body fat and distribution, a condition not investigated in this study. These findings seem to be consistent with other studies which found that high levels of triglyceride were more positively correlated with body fat than with changes in blood pressure [21, 22].

We discovered a marked increase in levels of cholesterol and triglycerides in diabetic and nondiabetic COVID-19-infected patients. Creatinine, GPT, ALP, and GOT values were shown to be higher than the normal range in some COVID-19-infected patients. The most notable comorbidities with COVID-19 in our study were diabetes (23%) and hypertension (14%). Coronaviruses bind to their target cells via angiotensin-converting enzyme 2 (ACE2), which is widely expressed in the kidney, intestine, and epithelial cells of the lung [23]. It has also been demonstrated that the expression of ACE2 is markedly upregulated in patients with diabetes which would promote the infection with COVID-19 [24, 25].

Several epidemiological data have shown no substantial correlation between smoking and disease severity in patients with COVID-19 [26–28]. In contrast, Leung et al. reported that smokers showed an upregulation of ACE2 gene expression than nonsmokers which facilitated COVID-19 infection [29]. When we analyzed the data according to smoking habits, elevated serum levels of triglyceride, creatinine, GPT, ALP, GOT, and urea were shown in few patients. Besides that, the concentrations of magnesium, phosphorus, and calcium were decreased in other patients. Interestingly, potassium levels showed significant differences when comparing smokers with nonsmoker patients. It has been shown that smoking habits might have induced alterations in potassium levels [30, 31]. However, the link between smoking and potassium levels has not been well studied. In conclusion, our results suggest several potential biochemical indexes change in patients with COVID-19 and that certain patient and clinical characteristics may influence these indexes.

## Figures and Tables

**Table 1:** Biochemical characteristics of patients with COVID-19 (*N* = 100).

ID	Ca	Na	K	Cl	Mg	P	Urea	Crea.	UA	Chol.	Tri	HDL	GOT	GPT	ALK	TSB	D	BP	S	C	G	Age
1	1.97	139	4.1	99	1.9	3.2	29	0.7	4.8	178	134	32	26	32	178	0.3	No	No	No	No	F	35
2	2.16	141	4	100	1.9	2.5	28	0.6	5.2	160	125	36	45	43	206	0.4	No	No	No	No	F	19
3	2.42	138	3.9	99	1.8	2.6	30	0.8	4.4	210	166	31	34	26	167	0.3	No	No	No	No	M	45
4	2.46	144	3.9	102	2	2.7	29	0.7	4.2	156	156	43	32	31	156	0.2	Yes	No	No	No	F	29
5	2.33	137	4	101	1.8	2.8	33	0.8	4.9	188	145	45	23	32	166	0.2	No	No	No	No	F	33
6	2.4	138	3.8	99	2.1	3.3	32	0.8	3.9	149	234	32	19	23	145	0.3	No	No	No	No	F	20
7	2.25	140	4.1	98	2	2.6	41	1.3	5.6	154	198	45	40	46	345	0.7	No	No	No	No	M	41
8	2.3	143	4.4	99	1.2	3.3	37	1.2	5.1	177	198	47	33	29	189	0.6	No	No	No	No	F	38
9	2.43	141	4.1	99	1.9	3.5	27	0.6	3.8	182	167	51	24	27	156	0.3	No	Yes	No	No	M	19
10	2.5	144	4.2	98	1.9	3.2	38	1.4	4.6	163	156	46	28	37	176	0.4	Yes	No	No	No	F	44
11	2.35	145	4	100	1.8	2.9	36	1.2	4.8	148	167	39	19	26	145	0.3	No	No	No	No	M	39
12	2.39	143	4.3	99	2	4.2	29	0.7	3.8	198	233	37	21	31	148	0.2	No	No	No	No	F	19
13	2.42	144	3.9	98	2.1	3.6	29	0.7	3.7	202	256	35	34	41	198	0.5	No	Yes	No	No	F	18
14	2.37	148	4	101	1.9	3.2	28	0.6	4	139	167	47	45	54	356	0.8	No	No	No	No	F	23
15	2.32	141	3.8	102	2.1	2.5	27	0.6	3.8	168	198	48	44	54	398	0.8	No	No	No	No	M	27
16	2.2	142	4.1	99	1.9	2.6	30	0.8	4.1	190	216	36	38	35	189	0.7	Yes	No	No	No	F	22
17	2.2	139	4	98	1.8	2.7	30	0.9	4.5	175	198	51	37	35	169	0.4	No	No	No	No	F	30
18	2.19	137	3.9	99	2	2.8	35	1	5.5	167	189	44	25	27	144	0.3	No	No	No	No	M	52
19	2.31	139	3.9	100	2.2	3.3	29	0.6	3.7	185	215	33	27	37	154	0.4	No	No	No	No	F	42
20	2.5	141	4	101	2.1	2.6	29	0.8	4.2	172	189	46	29	40	189	0.7	No	Yes	Yes	No	M	34
21	2.1	138	3.8	102	1.9	3.3	35	1	5.4	167	177	47	39	43	188	0.5	No	No	No	No	F	62
22	2	144	4.1	103	1.9	3.5	44	1.3	5.7	144	155	51	47	54	349	0.8	No	No	No	Yes	M	23
23	2.29	137	4.4	102	1.8	3.2	29	0.8	4	136	145	40	78	86	412	0.9	No	No	Yes	No	M	36
24	2.52	138	4.1	101	2	2.9	28	0.6	3.6	184	199	50	45	54	377	1	Yes	No	No	No	F	51
25	2.37	140	4.2	99	1.8	4.2	30	0.8	4.2	156	178	47	37	38	190	0.6	No	No	No	Yes	F	28
26	1.97	143	4	99	2.1	3.6	45	1.6	5.6	165	197	44	45	44	336	0.9	No	Yes	No	No	M	74
27	2.16	141	4.3	100	2	3.2	49	1.7	5.9	176	195	51	67	81	367	1.1	Yes	No	No	No	F	63
28	2.42	144	3.9	99	1.2	2.5	30	0.8	4.4	157	176	45	24	26	144	0.3	No	No	Yes	No	M	22
29	2.46	145	4	102	1.9	2.6	40	1.3	6	134	154	42	26	31	145	0.3	Yes	No	No	No	F	54
30	2.33	143	3.8	101	1.9	2.7	44	1.4	5.7	155	188	39	17	23	136	0.2	No	No	No	No	M	37
31	2.4	144	4.1	99	1.8	2.8	30	0.8	4.3	146	165	36	19	22	127	0.2	No	Yes	No	No	F	25
32	2.25	148	4	98	2	3.3	31	0.9	4.4	181	241	40	34	28	134	0.3	No	No	No	No	F	17
33	2.3	141	3.9	99	2.1	2.6	28	0.7	3.8	155	186	45	22	19	122	0.3	No	No	No	Yes	F	20
34	2.43	142	3.9	99	1.9	3.3	29	0.8	4.6	184	216	54	37	35	149	0.4	No	No	No	No	M	55
35	2.5	139	4	98	2.1	3.5	32	0.9	5	194	216	34	35	38	178	0.5	Yes	No	No	No	M	46
36	2.35	137	3.8	100	1.9	3.2	27	0.6	3.5	176	187	44	27	29	138	0.3	No	No	No	No	F	36
37	2.39	139	4.1	99	1.8	2.9	33	0.9	4.8	155	167	38	44	49	352	0.7	No	No	No	No	M	34
38	2.42	141	4.4	98	2	4.2	34	0.9	5.2	172	190	60	28	27	134	0.4	Yes	No	No	No	F	18
39	2.37	138	4.1	101	2.2	3.6	41	1.2	5.9	146	176	41	19	28	113	0.3	No	No	No	No	F	43
40	2.32	144	4.2	102	2.1	3.2	35	1	6.1	186	209	38	36	39	177	0.5	No	No	No	No	M	55
41	2.2	137	4	99	1.9	2.5	29	0.7	3.7	167	178	51	41	44	190	0.9	Yes	No	No	No	M	26
42	2.2	138	4.3	98	1.9	2.6	38	1.4	5.9	154	189	44	23	21	123	0.3	No	No	Yes	No	M	65
43	2.19	140	3.9	99	1.8	2.7	28	0.6	3.5	187	216	43	24	23	127	0.3	Yes	No	No	No	F	26
44	2.31	143	4	100	2	2.8	36	1.1	4.7	213	256	34	16	27	161	0.3	No	No	Yes	No	F	44
45	2.5	141	3.8	101	1.8	3.3	29	0.7	4.4	167	178	53	25	34	166	0.4	No	No	No	No	F	34
46	2.1	144	4.1	102	2.1	2.6	28	0.7	3.7	223	267	43	56	68	423	0.8	No	No	Yes	No	M	22
47	2	145	4	103	2	3.3	44	1.5	6.2	234	255	41	36	43	412	0.9	Yes	No	Yes	No	M	31
48	2.29	143	3.9	102	1.2	3.5	30	0.8	4.9	145	198	42	31	28	185	0.4	No	No	No	No	F	33
49	2.52	144	3.9	101	1.9	3.2	51	1.8	6.6	166	189	34	25	31	136	0.5	No	Yes	Yes	No	M	48
50	2.37	148	4	99	1.9	2.9	29	0.7	4	199	231	43	22	26	124	0.4	No	No	No	No	F	29
51	1.97	141	3.8	99	1.8	4.2	34	0.9	4.3	211	223	32	16	19	123	0.3	Yes	No	Yes	No	M	46
52	2.16	142	4.1	100	2	3.6	39	1.5	5.8	225	245	37	43	55	369	0.8	No	No	No	No	F	70
53	2.42	139	4.4	99	2.1	3.2	37	1.3	5.4	157	156	34	26	28	116	0.4	No	No	No	Yes	M	24
54	2.46	137	4.1	102	1.9	2.5	29	0.8	4.6	145	175	38	53	68	390	0.7	No	No	Yes	No	M	38
55	2.33	139	4.2	101	2.1	2.6	27	0.6	3.9	187	190	42	23	19	128	0.3	Yes	No	No	No	F	52
56	2.4	141	4	99	1.9	2.7	26	0.6	3.7	162	134	32	21	21	118	0.3	No	No	No	No	F	36
57	2.25	138	4.3	98	1.8	2.8	33	1	5.1	161	145	36	16	25	143	0.4	No	No	Yes	No	M	66
58	2.3	144	3.9	99	2	3.3	52	1.7	6.4	209	246	35	18	26	136	0.3	Yes	No	No	No	F	64
59	2.43	137	4	99	2.2	2.6	36	0.9	4.8	172	147	44	15	21	128	0.3	No	No	Yes	No	M	17
60	2.5	138	3.8	98	2.1	3.3	26	0.7	4	191	210	36	34	38	168	0.6	No	No	No	No	F	23
61	2.35	140	4.1	100	1.9	3.5	29	0.8	4.7	226	233	29	26	33	156	0.5	No	No	Yes	No	M	45
62	2.39	143	4	99	1.9	3.2	30	0.7	4.1	179	189	34	56	71	355	0.8	Yes	No	No	No	F	30
63	2.42	141	3.9	98	1.8	2.9	33	0.9	5.2	156	167	45	67	76	423	0.9	No	No	No	No	F	27
64	2.37	144	3.9	101	2	4.2	32	0.8	4.8	188	198	38	30	33	187	0.7	No	No	Yes	No	M	28
65	2.32	145	4	102	1.8	3.6	31	0.9	5.7	159	145	50	33	36	197	0.5	No	No	No	Yes	M	37
66	2.2	143	3.8	99	2.1	3.2	35	1.2	6.6	190	189	46	24	28	127	0.4	Yes	No	No	No	F	49
67	2.2	144	4.1	98	2	2.5	29	0.7	4.9	209	215	33	26	32	123	0.3	No	No	Yes	No	M	26
68	2.19	148	4.4	99	1.2	2.6	29	0.7	4.3	188	190	43	35	34	154	0.5	No	No	No	No	F	24
69	2.31	141	4.1	100	1.9	2.7	28	0.6	3.8	167	165	42	37	40	198	0.8	Yes	No	Yes	No	M	56
70	2.5	142	4.2	101	1.9	2.8	27	0.6	3.6	156	124	38	21	23	121	0.3	No	No	Yes	No	M	45
71	2.1	139	4	102	1.8	3.3	31	0.9	5.3	211	212	48	22	26	122	0.3	No	No	No	No	M	36
72	2	137	4.3	103	2	2.6	30	0.8	4.7	241	312	51	39	36	178	0.4	Yes	No	No	No	F	21
73	2.29	139	3.9	102	2.1	3.3	29	0.7	4.4	154	133	34	34	31	151	0.3	No	No	No	No	F	28
74	2.52	141	4	101	1.9	3.5	40	1.4	6.1	176	127	36	33	30	154	0.3	No	No	Yes	No	M	52
75	2.37	138	3.8	99	2.1	3.2	47	1.6	6.4	133	123	36	36	32	145	0.5	No	No	No	No	F	46
76	1.97	144	4.1	99	1.9	2.9	28	0.8	4.4	184	199	44	28	35	147	0.4	No	Yes	Yes	No	M	37
77	2.16	137	4	100	1.8	4.2	39	1.3	5.4	178	188	46	19	21	114	0.2	No	No	No	No	F	57
78	2.42	138	3.9	99	2	3.6	38	1.4	5.8	167	156	52	28	33	123	0.3	No	No	Yes	No	M	60
79	2.46	140	3.9	102	2.2	3.2	27	0.6	3.5	198	200	54	37	42	165	0.4	Yes	No	No	Yes	M	21
80	2.33	143	4	101	2.1	2.5	43	1.5	6.2	195	198	39	44	48	214	0.7	No	Yes	Yes	No	M	48
81	2.4	141	3.8	99	1.9	2.6	36	1	6	155	112	40	35	36	177	0.6	No	No	No	No	F	40
82	2.25	144	4.1	98	1.9	2.7	29	0.7	4.8	210	243	38	27	23	121	0.3	No	No	Yes	No	M	26
83	2.3	145	4.4	99	1.8	2.8	31	0.8	4.4	145	145	34	24	19	126	0.3	No	No	Yes	No	M	41
84	2.43	143	4.1	99	2	3.3	32	0.9	4.9	178	167	41	23	15	144	0.4	No	No	No	No	F	67
85	2.5	144	4.2	98	1.8	2.6	29	0.7	5.1	155	122	35	17	18	155	0.5	No	No	No	No	F	38
86	2.35	148	4	100	2.1	3.3	43	1.5	6.3	182	190	44	37	38	156	0.5	No	Yes	No	No	F	23
87	2.39	141	4.3	99	2	3.5	33	0.9	5.5	196	197	45	41	47	213	0.7	No	No	Yes	No	M	25
88	2.42	142	3.9	98	1.2	3.2	29	0.8	5.8	228	265	35	40	45	245	0.9	Yes	Yes	No	No	F	32
89	2.37	139	4	101	1.9	2.9	29	0.7	4.9	128	133	36	69	82	434	1.1	No	No	No	No	F	29
90	2.32	137	3.8	102	1.9	4.2	31	0.9	5.9	168	178	38	71	93	390	1.2	No	No	No	No	F	19
91	2.2	139	4.1	99	1.8	3.6	30	0.8	4.5	190	176	48	23	25	134	0.3	No	No	Yes	No	F	25
92	2.2	141	4	98	2	3.2	26	0.6	4.1	205	234	53	27	31	143	0.4	No	Yes	No	No	F	22
93	2.19	138	3.9	99	2.1	2.5	30	0.8	5.2	213	213	29	28	24	134	0.2	Yes	Yes	No	No	M	16
94	2.31	144	3.9	100	1.9	2.6	36	1.2	5.9	189	199	35	16	17	114	0.3	No	No	No	No	F	31
95	2.5	137	4	101	2.1	2.7	29	0.7	3.8	179	189	34	15	21	115	0.3	No	No	Yes	No	M	40
96	2.1	138	3.8	102	1.9	2.8	28	0.6	3.9	135	134	36	24	26	134	0.5	Yes	No	No	No	F	41
97	2	140	4.1	103	1.8	3.3	38	0.8	4.7	150	156	32	33	34	157	0.4	No	Yes	No	No	M	24
98	2.29	143	4.4	102	2	2.6	40	1.3	6.1	182	187	48	22	28	156	0.6	No	No	Yes	No	M	55
99	2.52	141	4.1	101	2.2	3.3	55	1.7	6.5	173	156	45	31	34	176	0.6	No	No	No	No	F	59
100	2.37	144	4.2	99	2.1	3.5	37	1.3	5.8	197	145	38	37	39	186	0.5	Yes	Yes	No	No	M	41

Ca: calcium; Na: sodium; K: potassium; Cl: chloride; Mg: magnesium; P: phosphorus; Crea.: creatinine; Chol.: cholesterol; Trig: triglyceride; HDL: high-density lipoprotein; GOT: aspartate aminotransferase; GPT: alanine aminotransferase; ALK: alkaline phosphatase; TSB; total bilirubin; D: diabetic; B: blood pressure; S: smoking; C: cancer; G: gender.

**Table 2:** Comparison summary of biochemical parameters among patients with COVID-19 (*N* = 100).

Parameters	Mean ± SD

Ca, mmol/L	2.31 ± 0.15
Na, mmol/L	141.25 ± 2.94
K, mmol/L	4.04 ± 0.17
Cl, mmol/L	99.92 ± 1.47
Mg, mg/dL	1.92 ± 0.2
P, mg/dL	3.09 ± 0.47
Urea, mg/dL	33.27 ± 6.3
Creatinine, mg/dL	0.94 ± 0.32
Uric acid, mg/dL	4.86 ± 0.87
Cholesterol, mg/dL	176.18 ± 24.68
Triglyceride, mg/dL	186.56 ± 37.67
HDL, mg/dL	41.26 ± 6.62
GOT, U/L	32.28 ± 12.86
GPT, U/L	36 ± 15.59
ALK, U/L	192.72 ± 90.59
TSB, mg/dL	0.49 ± 0.24

## Data Availability

All data are included in the supplementary materials.

## Supplementary Materials

Supplementary Materials Overview of the clinical information, age, gender, and biochemical parameters for the 100 participants in the study.
